# Effect of Bioactive Glasses and Basic Fibroblast Growth Factor on Dental Pulp Cells

**DOI:** 10.3390/jfb14120568

**Published:** 2023-12-18

**Authors:** Ayako Washio, Olivia Kérourédan, Yasuhiko Tabata, Shoichiro Kokabu, Chiaki Kitamura

**Affiliations:** 1 Division of Endodontics and Restorative Dentistry, Department of Oral Functions, Kyushu Dental University, 2-6-1 Manazuru, Kokurakita-ku, Kitakyushu 803-8580, Japan; r06kitamura@fa.kyu-dent.ac.jp; 2 National Institute of Health and Medical Research (INSERM), U1026 BIOTIS, University of Bordeaux, 146 Rue Léo Saignat, 33076 Bordeaux, France; olivia.kerouredan@u-bordeaux.fr; 3 Laboratory of Biomaterials, Department of Regeneration Science and Engineering, Institute for Frontier Life and Medical Sciences, Kyoto University, 53 Kawara-cho Shogoin, Sakyo-ku, Kyoto 606-8507, Japan; yasuhiko@infront.kyoto-u.ac.jp; 4 Division of Molecular Signaling and Biochemistry, Kyushu Dental University, 2-6-1 Manazuru, Kokurakita-ku, Kitakyushu 803-8580, Japan; r14kokabu@fa.kyu-dent.ac.jp

**Keywords:** bioactive glasses, basic fibroblast growth factor, dental pulp cells

## Abstract

Ideal regeneration of hard tissue and dental pulp has been reported with the use of a combination of bioactive glass and basic fibroblast growth factor (bFGF). However, no previous study has investigated the molecular mechanisms underlying the processes induced by this combination in dental pulp cells. This study aimed to examine the cellular phenotype and transcriptional changes induced by the combination of bioactive glass solution (BG) and bFGF in dental pulp cells using phase-contrast microscopy, a cell counting kit-8 assay, alkaline phosphatase staining, and RNA sequence analysis. bFGF induced elongation of the cell process and increased the number of cells. Whereas BG did not increase ALP activity, it induced extracellular matrix-related genes in the dental pulp. In addition, the combination of BG and bFGF induces gliogenesis-related genes in the nervous system. This is to say, bFGF increased the viability of dental pulp cells, bioactive glass induced odontogenesis, and a dual stimulation with bioactive glass and bFGF induced the wound healing of the nerve system in the dental pulp. Taken together, bioactive glass and bFGF may be useful for the regeneration of the dentin-pulp complex.

## 1. Introduction

Dental caries or traumatic injuries may result in dental pulp exposure [1,2]. Direct pulp capping induces wound healing in teeth with pulp exposure via the formation of hard tissue (reparative dentin), which can protect the remaining dental pulp [3,4]. However, the formation of reparative dentin following direct pulp capping may reduce the size of the pulp chamber, blood flow, and vitality of the dental pulp [5,6]. Additionally, the pulp capping materials we currently use have poor adhesion to the dentin surface [7,8]. It is suggested that the limitation of pulp capping induces the fragility of dental pulp or second caries and can lead to pulp loss, tooth loss, and deterioration in the quality of life of the patient. In this regard, regenerative therapy, which induces the regeneration of the dental pulp with the formation of a dentin layer in the area of dentin defects, is ideal.

Basic fibroblast growth factor (bFGF) plays an essential role in tooth development [9,10], the proliferation of dental pulp stem cells [11], and odontoblast differentiation [12,13]. Gelatin hydrogels gradually release bFGF during biodegradation [14]. Thus, the duration of the release of bFGF with the use of gelatin hydrogels is greater than that observed following the injection of free bFGF [14,15]. Controlled release of bFGF from the implanted gelatin hydrogel induces regeneration of the dental pulp with the formation of a dentin-like hard tissue layer over the dentinal defect above the dental pulp [16,17]. In contrast, uncontrolled release of bFGF induces reparative dentin formation in the residual dental pulp [16]. These findings suggest that the controlled release of bFGF is effective for the regeneration of hard tissues and dental pulp. However, the regenerated dentin-like hard tissue differs from natural dentin, especially in terms of structure. The imperfection in the regenerated dentin-like hard tissue may be attributed to the inability of the implanted scaffold to induce proper mineralization via the crystallization of hydroxyapatite.

Bioactive glass is a subset of bioactive ceramics that can react with physiological fluids to form tenacious bonds with the bone through the formation of bone-like hydroxyapatite layers and the biological interaction of collagen with the material surface [18]. Our previous study demonstrated that gelatin hydrogel sponges capable of controlled release of bFGF-incorporated bioactive glass particles (BG-bFGF gel) were fabricated to regenerate highly calcified dentin-like hard tissues [19]. We also revealed the mechanical and chemical functionality of BG-bFGF gel as scaffolds capable of controlled release of bFGF and the induction of hydroxyapatite crystallization [19]. These data suggest that our fabricated BG-bFGF gel has important biomaterial applicability in wound healing and the regeneration of complex tissues such as dental pulp and dentin.

Previous studies have demonstrated that bioactive glasses can induce odontogenic differentiation of dental pulp cells in vitro, promote dentin-pulp complex regeneration in vivo [20,21], and regulate the extracellular matrix microenvironment via paracrine actions [22]. Thus, it can be hypothesized that the combination of bioactive glass and bFGF can be used to achieve the ideal regeneration of hard tissue and dental pulp, both in terms of biological outcomes. However, no previous study has elucidated the molecular mechanisms underlying the processes induced by the combination in dental pulp cells. Therefore, this study aimed to examine the cellular phenotype and transcriptional changes induced by the combination of bioactive glass and bFGF in dental pulp cells.

## 2. Materials and Methods

### 2.1. Materials

The materials used in this study have been described in previous studies [19]. Bioactive glass particles (BGP; average granule diameter of 4 μm, molar ratio of Ca/P = 45.6, mass ratio of Ca/P = 47.4) comprise CaO, SiO_2_, and P_2_O_5_ (Nippon Shika Yakuhin Co., Ltd., Yamaguchi, Japan). Reagent-grade SiO_2_, Ca (PO_3_)_2_, and CaCO_3_ were mixed together, and the mixture was heated at 1500 °C to obtain melts. The melts were cooled and crushed subsequently to obtain BGP. X-ray diffraction was used to confirm that BGP was not in a crystalline phase. Human recombinant bFGF was purchased from PeproTech Inc. (Cranbury, NJ, USA).

### 2.2. Preparation of Liquid Extract from BGP

**Figure 1:**
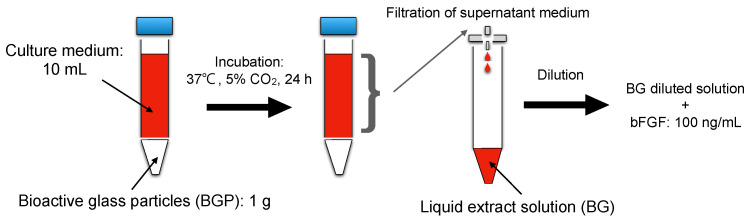
Schematic representation of preparing liquid extract from.

A liquid extract of BGP was prepared according to ISO 10993-5 (extraction ratio: 0.1 g/mL) [23]. In brief, 10 mL of culture medium supplemented with 5% heat-inactivated fetal bovine serum (FBS) was added to 1.0 g of BGP in a centrifuge tube (15 mL). Culture medium supplemented with 5% FBS was used as the control. The tube was then incubated at 37 °C in a humidified atmosphere of 5% CO_2_ for 24 h. The medium was collected and filtered using 0.2 μm sterile syringe filters (Merck, Darmstadt, Germany) to obtain a liquid extract (BG) of BGP. BG was diluted from 1/2 to 1/16 in the culture medium (Figure 1).

### 2.3. Cell Culture

A rat clonal dental pulp cell line (KN-3) [24]. The KN-3 cell line was established in our laboratory, led by Dr. Kitamura. It was maintained in alpha-modified Eagle’s medium (a-MEM) (Invitrogen Life Technology, Carlsbad, CA, USA) containing 10% FBS, 100 mg/mL of streptomycin, and 100 U/mL of penicillin and subsequently cultured in 100 mm dishes at 37 °C in a humidified atmosphere of 5% CO_2_ [24].

### 2.4. Morphological Analysis

The morphological changes in KN-3 cells treated with BG and bFGF were analyzed. For this purpose, the cells (1.0 × 10^4^/well) were sub-cultured in 24-well plates for 4 h and then treated with BG (1/2 dilution) and bFGF (100 ng/mL). The morphology of the cells was observed using phase-contrast microscopy after 48 h. The observation was performed in a double-blind fashion by three people who did not know the critical aspects of the experiment.

### 2.5. Cell Viability Analysis

Cell viability of KN-3 cells treated with BG and bFGF was evaluated according to ISO 10993-5 (2009) standards by measuring the amount of formazan dye generated by dehydrogenases in the cells using a cell counting kit-8 (CCK-8) assay (DOJINDO, Kumamoto, Japan) as per a protocol based on the manufacturer’s instructions [23]. Serially diluted BG and bFGF (100 ng/mL) were added to the cells (3.0 × 10^4^/well) cultured in 96-well plates for 4 h. CCK-8 solution (10 μL) was added to each well of the plate after 48 h, and the optical density (OD) at 450 nm was measured using an iMark microplate reader (Bio-Rad, Hercules, CA, USA) after incubation at 37 °C for 3 h. Statistically significant differences were determined using one-way analysis of variance (ANOVA) combined with a *t*-test. The data are expressed as the mean ± SD. Furthermore, *p*-values lower than 0.05 were regarded as significant (* *p* < 0.05).

### 2.6. Alkaline Phosphatase Staining

Alkaline phosphatase (ALP) staining was performed to examine the differentiation of KN-3 cells treated with BG and bFGF. BG (1/2 dilution) and bFGF (100 ng/mL) were added to cells (1.0 × 10^5^/well) sub-cultured in 24-well plates for 24 h. The cells were fixed with 4% paraformaldehyde (FUJIFILM Wako Pure Chemical, Osaka, Japan; PFA) in PBS for 10 min at room temperature after 7 days and stained using a nitro-blue tetrazolium chloride/5-bromo-4-chloro-3′-indolylphosphate p-toluidine salt stock solution (Sigma-Aldrich, Saint Louis, MO, USA) as per the manufacturer’s instructions [19].

### 2.7. RNA-Seq Analysis

RNA-Seq analysis was performed to examine the transcriptional changes induced by BG or the combination of BG and bFGF (BG + bFGF). BG (1/2 dilution) or BG + bFGF (100 ng/mL) was added to cells (1.0 × 10^5^/well) sub-cultured in 6-well plates for 4 h. The total RNA was isolated from the cells using the FastGene TM RNA Basic Kit (Nippon Genetics, Tokyo, Japan) after 48 h. The total RNA used for the preparation of RNA-seq libraries and sequencing was provided by Macrogen Japan (Tokyo, Japan). The RNA-seq libraries were prepared using a TruSeq stranded mRNA LT Sample Prep Kit (Illumina Inc., San Diego, CA, USA), and the libraries were sequenced using NovaSeq6000 (Illumina Inc., San Diego, CA, USA).

## 3. Results

### 3.1. Morphology of the KN-3 Cells Treated with BG and bFGF

**Figure 2:**
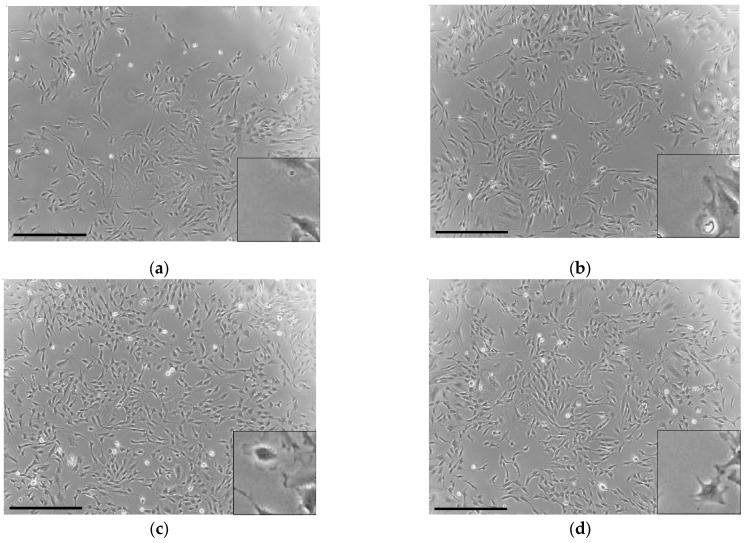
Phase-contrast microscopic photographs depicting the morphology of the KN-3 cells: (**a**) control (culture medium without BG and bFGF); (**b**) BG-treated cells; (**c**) bFGF-treated cells; (**d**) BG + bFGF-treated cells. Bar: 500 μm. The bottom-right images show the magnified versions of each image.

The cells were elongated and spindle-shaped in the control group (Figure 2a). BG was found to have no effect on the morphology of the cells (Figure 2b). In contrast, bFGF was found to induce elongation of the cell process (Figure 2c,d).

### 3.2. Viability of the KN-3 Cells Treated with BG and bFGF

**Figure 3:**
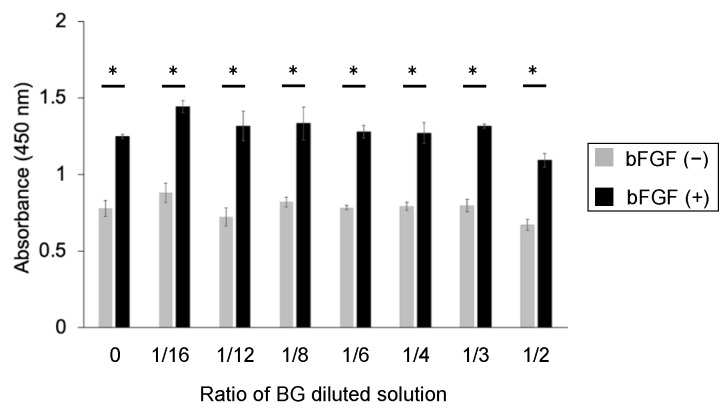
CCK-8 assay of untreated KN-3 cells and those treated with BG and bFGF. Gray bar graph: the data of BG-treated KN-3 cells without bFGF. Black bar graph: the data of BG + bFGF-treated KN-3 cells. X-axis: the ratio of BG to culture medium. The data are shown as the mean ± standard error and were obtained from triplicate cultures for each representative experiment. * *p* < 0.05.

No significant differences were observed in the number of BG-treated cells compared with control; however, bFGF was found to significantly increase the number of KN-3 cells compared with control and BG-treated cells (*p* < 0.05, Figure 3).

### 3.3. ALP Staining of the KN-3 Cells Treated with BG and bFGF

**Figure 4:**
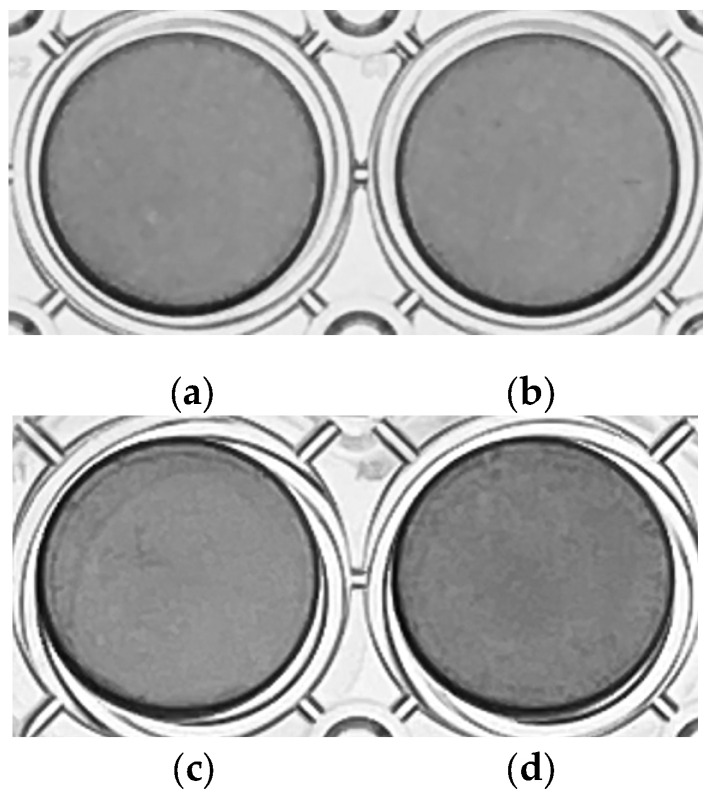
ALP staining of untreated KN-3 cells and those treated with BG and bFGF. The images were obtained from triplicate cultures for each representative experiment. (**a**) control; (**b**) BG-treated cells; (**c**) bFGF-treated cells; (**d**) BG + bFGF-treated cells.

BG and bFGF were found to have no effect on odontoblast differentiation. No significant differences were qualitatively observed in the ALP activity of BG-treated, bFGF-treated, and BG + bFGF-treated cells (Figure 4).

### 3.4. Overview of Differentially Expressed Genes in the Control and Treated Groups

**Figure 5:**
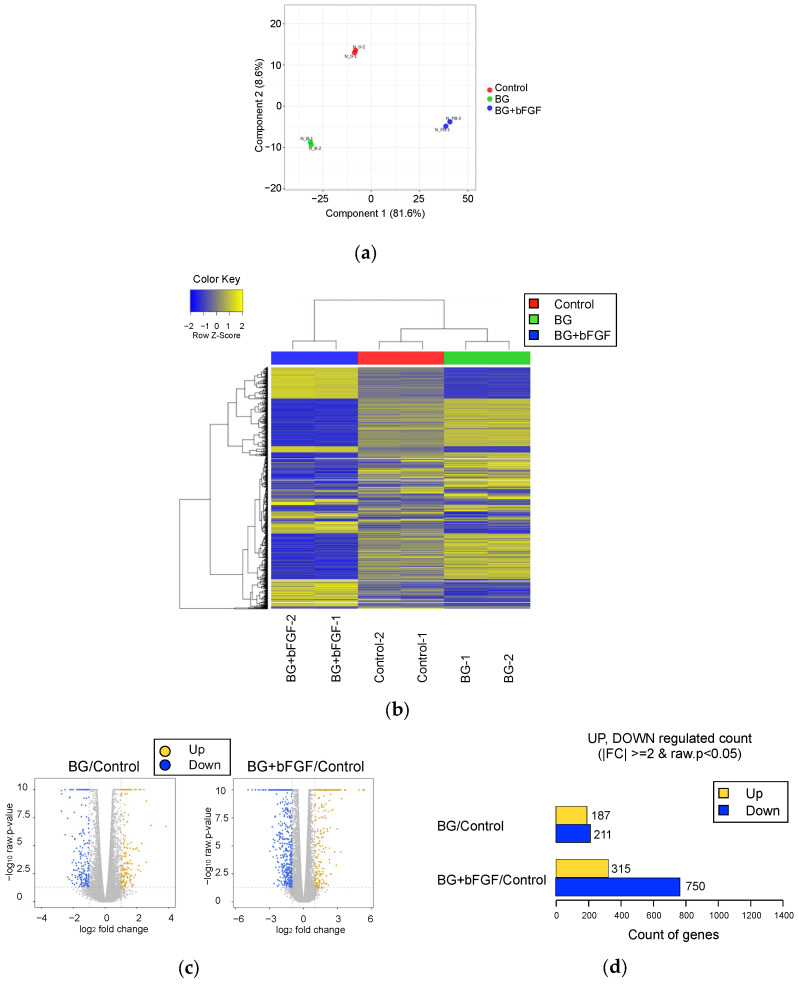
Gene expression in the control and treated groups. (**a**) PCA of normalized RNA-seq read counts. Each independent replicate is clustered for each group; however, a higher dispersion is observed in the BG and BG + bFGF groups than that in the Control group. (**b**) Heat map of the one-way hierarchical clustering of 2282 genes using Z-score for normalized value (log2-based). Each column represents a group, and each row represents a gene. Clustering was performed using iDEP with edgeR log transformations of the read counts. Hierarchical clustering is illustrated using the average linkage method with correlation distance. Color coding is based on edgeR log-transformed read counts. The color key indicates the Z-scores, which are displayed as relative values for all tiles within the samples. The blue and yellow bars indicate the lowest level and the highest level of expression, respectively. (**c**) Volcano plot of the expression level of the BG and BG + bFGF groups compared with that of the Control group. (**d**) The number of upregulated and downregulated genes based on the fold change and *p*-value of the BG or BG + bFGF groups compared with that of the Control group.

A comprehensive gene expression analysis was performed using RNA-seq. Sample-level quality control was performed using principal component analysis (PCA), wherein the normalized read counts were used to visualize the variation among the treated groups and confirm the similarity of each sample receiving the same treatment (Figure 5a). Global transcriptional changes among the samples were evaluated using a heatmap and dendrogram showing hierarchical clustering under these conditions (Figure 5b). The first principal component accounted for 81.6% of the total variability, which corresponded to the reference sequence-specific variance, whereas the subsequent principal components accounted for 8.6%. The hierarchical clustering of the groups reiterated the relationships observed during PCA. The cells treated with BG or BG + bFGF exhibited different gene expression patterns.

The log2 fold change and *p*-value obtained from the comparison between the two groups plotted as a volcano plot were used to visualize the direction, magnitude, and significance of changes in gene expression of the BG and BG + bFGF groups compared with those of the control group (Figure 5c). The most upregulated genes were observed toward the right, whereas the most downregulated genes were observed toward the left. The most statistically significant genes were observed toward the top. Figure 5d shows the number of genes exhibiting differential expression in each group using up- and downregulated counts by fold change and *p*-value. Compared with the control group, significant upregulation of 187 genes and downregulation of 211 genes were observed in the BG group. Compared with the control group, significant upregulation of 315 genes and downregulation of 750 genes were observed in the BG + bFGF group.

### 3.5. Analysis of Gene Ontology and Gene Expression in the Control and Treated Groups

**Figure 6:**
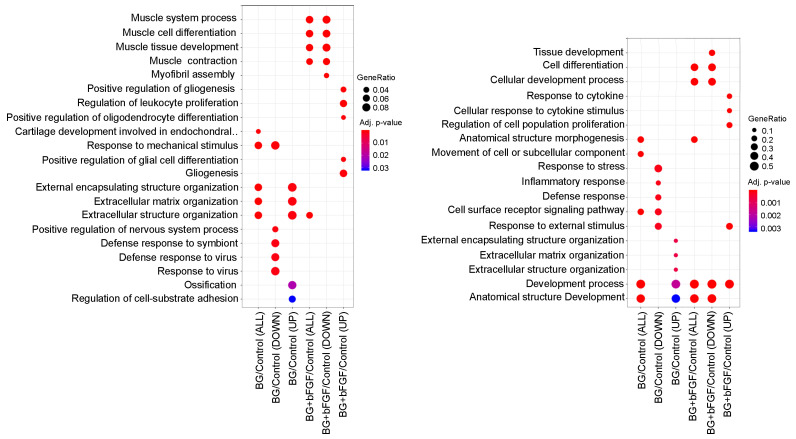
GO enrichment analysis of DEGs A dot plot depicts the upregulated and downregulated GO terms of biological processes. The color of the dots represents the *p*-value of the GO enrichment significance, and the size of the dot represents the gene ratio based on gene count in the GO term.

The transcriptome and gene ontology (GO) enrichment of differentially expressed genes (DEGs) were analyzed to identify the types of characteristic genes in the BG and BG + bFGF groups (Figure 6). BG/Control (UP) and BG/Control (DOWN) revealed that GO and genes for DEGs were up- and downregulated, respectively, in the BG group compared with that in the control group. Differentially upregulated genes in the BG/Control groups were enriched in GO terms such as “external encapsulating structure organization”, “extracellular matrix organization”, “extracellular structure organization”, “ossification”, and “regulation of cell-substrate adhesion”. In contrast, differentially downregulated genes in the BG/Control groups were enriched in GO terms, such as “response to stress”, “inflammatory response”, “defense response”, and “response to external stimulus”. BG + bFGF/Control (UP) and BG + bFGF/Control (DOWN) revealed that GOs and genes for DEGs were upregulated and downregulated, respectively, in the BG + bFGF group compared with those in the Control group. Differentially upregulated genes in the BG + bFGF/Control were enriched in GO terms such as “response to cytokine”, “gliogenesis”, and “regulation of cell population proliferation”. In contrast, the DEGs in the BG + bFGF/Control were enriched in GO terms, such as “muscle cell differentiation”. The differentially expressed genes extracted from the upregulated GO terms of BG/Control, which mainly included *Spock2*, *Abi3bp*, *Postn*, and *Fmod*, are shown in Table 1. Similarly, the extraction of differentially expressed genes in the upregulated GO terms of BG + bFGF/Control, which mainly included *Tenm4*, *Pparg*, *Tlr2*, and *Il33*, is shown in Table 2.

## 4. Discussion

This study evaluated the effects of BG and bFGF by examining the changes in the morphology, viability, and odontogenic differentiation of dental pulp cells. The present study revealed that BG had no adverse effects on the morphology or viability of dental pulp cells. These data are consistent with those of the previous study that used cementoblast-like, periodontal ligament-like, and osteoblast-like cells cultured with bioactive glass-containing biomaterials [25,26]. These results suggest that BG has no cytotoxic effects on dental pulp cells or periodontal tissue-related cells.

The function of the combination of BG and bFGF on the cell was analyzed using RNA-seq, which is a high-throughput sequencing method that provides insight into the transcriptome. This analysis is one of the most common applications for studying differential gene expression [27]. RNA-Seq analysis identified a very large number of genes (2282 DEGs) among the three groups. Hierarchical clustering with the dendrogram in Figure 5 indicates a closer correlation between the BG and control groups, whereas the BG + bFGF group was observed to be more distant from the two groups.

**Figure 7:**
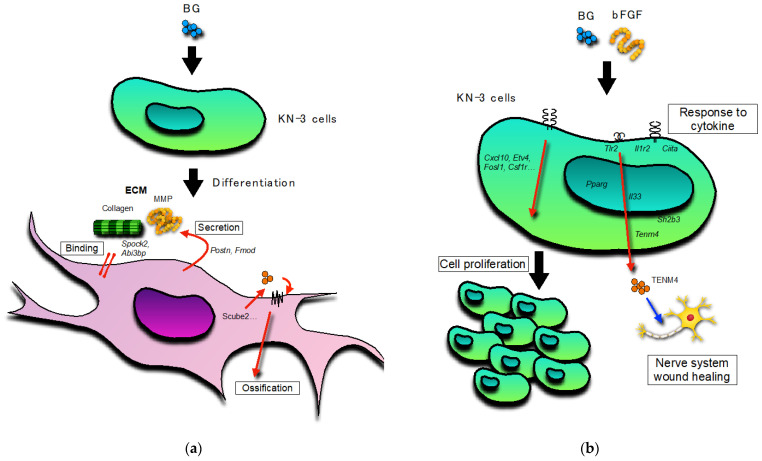
Schematic representation: (**a**) The leading role of BG-treated KN-3 cells; (**b**) The leading role of BG and bFGF-treated KN-3 cells. Black arrow: activation. Red arrow: induction. Blue arrow: stimulation.

BG, with or without bFGF, had no effect on the ALP activity in the KN-3 cells (Figure 4). In contrast, BG up-regulated extracellular matrix (ECM)-related GO terms and genes and cell differentiation-related GO terms and genes in KN-3 cells (Figure 6 and Table 1). Dental pulp cells participate in ECM remodeling through the synthesis of collagen and fibronectin and their degradation by matrix metalloproteinases [28,29]. A bioactive glass-containing dental material implanted into rat back subcutaneous tissue was found to upregulate the expression of ECM-related genes [30]. Furthermore, bioactive glass induces odontogenic differentiation and dentin formation in dental pulp cells and may serve as a potential material for pulp repair and dentin regeneration [20,31,32]. Previous studies have shown that *spock2* and *Abi3bp* encode proteins that bind with glycosaminoglycans to form ECM [33,34]. Moreover, *Fmod* was suggested to be related to the formation of ECM [35], whereas *Postn* was suggested to be the gene encoding a secreted ECM protein that regulates tissue development and regeneration, including wound healing [36]. Scube 2 plays an essential role in osteogenesis and bone homeostasis [37]. Therefore, it is suggested that BG induces the formation of ECM structures and the binding of cells to the ECM via the upregulation of *Spock2*, *Abi3bp*, *Fmod*, and *Postn*, which results in the induction of dentinogenesis in odontoblast-like cells via the upregulation of *Scube2* in this study (Figure 7).

Cells treated with bFGF with or without BG showed elongation and an increase in cell viability. These results were supported by the up-regulation of cell proliferation-related GO terms and genes and the significant upregulation of the BG + bFGF group compared with the control. Previous studies have reported that *Etv4* is transcriptionally regulated by bFGF-signals in many embryonic contexts [38,39,40,41,42]. The findings of the present study suggest that the viability of bFGF-treated dental pulp cells increased as a result of the upregulation of *Etv4* (Figure 7).

Treated dental pulp cells secrete neurotrophins and promote prominent neurite outgrowth toward the site of carious injury [43,44,45]. This study suggests that the BG + FGF-treated dental pulp cells positively regulate the growth and differentiation of glia in the dental pulp. Schwann cells are the principal glial cells of the peripheral nervous system. Suppression of neurodestructive M1 phenotype macrophages and maintenance of neuroprotective M2 phenotype macrophages by Schwann cells maintain the viability of highly innervated dental pulp [46]. A previous study reported that the proliferation of Schwann cells was predominantly promoted and apoptosis was predominantly inhibited when dental pulp stem cells and Schwann cells were co-cultured [47]. Thus, Schwann cells play a crucial role in maintaining pulpal integrity and sensory function during dentin-pulp regeneration. However, the underlying mechanisms have not yet been elucidated. In this study, BG + bFGF upregulated *Tenm4*, *Pparg*, *Tlr2*, *Il33*, and *Tnfrsf1b*. Tenm4 plays a key role in regulating neurite outgrowth and acts as a regulator of oligodendrocyte differentiation [48]. *Il-33*, which is present in the nuclei of epithelial cells and fibroblasts, is involved in wound healing [49]. Thus, the findings of the present study suggest that the activity of *Il-33* and secretion of *Tenm4* in the BG + bFGF-treated dental pulp cells play a role in wound healing in the intra-pulpal nerve system. Furthermore, the BG + bFGF-treated dental pulp cells showed high expression of genes such as receptors related to cytokine responses (*Il1r2*, *Il6r*, and *ciita*), suggesting that they respond to cell proliferation, differentiation, and pulp wound healing (Figure 7).

On the other hand, BG downregulated inflammation-related GO terms and genes in the dental pulp cells (Figure 6). Dental pulp cells secrete various cytokines and regulate the expression of various proinflammatory mediators in response to bacterial stimulation. Moreover, they participate in neurogenic inflammation and dental pulp wound healing and amplify the pulpal immune response in response to inflammatory mediators [50,51,52,53]. Thus, inflammatory reactions to the materials should not occur after the removal of the bacterial stimulation. The present study suggests that bioactive glass plays an anti-inflammatory role. In contrast, BG + bFGF downregulated extracellular myogenesis-related GO terms and genes in KN-3 (Figure 6). The dental pulp cells are capable of differentiating into osteoblasts and chondrocytes and switching their genetic program when co-cultured with murine myoblasts [54,55,56,57]. Previous studies indicate that dental pulp cells have muscle-specific genes that can be activated through myogenic fusion, confirming their multipotency. The findings of the present study suggest that KN-3 is a multipotent dental pulp cell, as it has hard tissue differentiation-related and muscle-related genes. Previous studies have reported that the upregulation of the FGF receptor-p38 pathway impairs the growth of skeletal muscle satellite cells [58,59]. The present study also showed that bFGF downregulates myogenesis-related genes. The effects of bioactive glasses on muscle regeneration have been investigated in recent years. The variation in the expression of muscle-related genes in different study groups indicates the importance of glass composition on muscle differentiation capacity in vitro [60,61,62]. This revealed that bioactive glass downregulated the expression of myogenesis-related genes.

Our present study suggests that bioactive glass and bFGF may be useful for not only dentin-pulp complex regeneration but also bone regeneration medicine because of the induction of cell viability and ossification. Needless to say, experiments to use bone cells are required. As an obvious limitation in our present study, we realize that the sampling time for RNA-seq analysis is only one. Therefore, we also realize that time-course analysis is needed in the future. Additionally, future work will focus on the in vivo experiment with our fabricated BG-bFGF gel incorporating bioactive glass particles capable of controlled release of bFGF. We predict that our fabricated BG-bFGF gel can be applied as an ideal biomaterial in wound healing and the regeneration of complex tissues such as dental pulp and dentin.

## 5. Conclusions

bFGF induced elongation of the cell process and increased the nucleus of living cells. Whereas bioactive glass did not increase ALP activity, it induced extracellular matrix-related genes. In addition, the combination of bioactive glass and bFGF induced gliogenesis-related genes in the nervous system. That is to say, bFGF increased the viability of dental pulp cells, bioactive glass induced odontogenesis, and a dual stimulation with bioactive glass and bFGF induced the wound healing of the nervous system. Taken together, bioactive glass and bFGF may be useful for the regeneration of the dentin-pulp complex.

## Figures and Tables

**Table 1:** Genes related to GO terms are upregulated by BG.

Classification	Go Term	Category	Gene Count	Gene Symbol
Extracellular matrix-related	External encapsulating structure organization	GO:0045229	11	*Spock2*, *Abi3bp*, *Postn*, *Olfml2a*, *Smpd3*, *Col6a4*, *Fmod*, *Gas2*, *Mmp15*, *Comp*, *Col11a2*
	Extracellular matrix organization	GO:0030198	11	*Spock2*, *Abi3bp*, *Postn*, *Olfml2a*, *Smpd3*, *Col6a4*, *Fmod*, *Gas2*, *Mmp15*, *Comp*, *Col11a2*
	Extracellular structure and organization	GO:0043062	11	*Spock2*, *Abi3bp*, *Postn*, *Olfml2a*, *Smpd3*, *Col6a4*, *Fmod*, *Gas2*, *Mmp15*, *Comp*, *Col11a2*
Cell adhesion-related	Regulation of cell-substrate adhesion	GO:0010810	7	*Limch1*, *Spock2*, *Abi3bp*, *Postn*, *Angpt2*, *Lims2*, *Tek*
Dentinogenesis -related	Ossification	GO:0001503	10	*Tek*, *Igf2*, *Bmp3*, *Col11a2*, *Gfra4*, *Comp*, *Scube2*, *Ifitm1*, *Wnt7b*, *Smpd3*

**Table 2:** Genes related to GO terms are upregulated by BG + bFGF.

Classification	Go Term	Category	Gene Count	Gene Symbol
Gliogenesis-related	Gliogenesis	GO:0042063	14	*Tenm4*, *Areg*, *Csf1r*, *Ccl2*, *Pparg*, *Tlr2*, *Adgrg6*, *Crb1*, *Il33*, *Adgrg1*, *Myc*, *Serpine2*, *Tnfrsf1b*, *Etv5*
	Positive regulation of gliogenesis	GO:0014015	8	*Tenm4*, *Pparg*, *Tlr2*, *Il33*, *Myc*, *Serpine2*, *Tnfrsf1b*, *Etv5*
	Positive regulation of glial cell differentiation	GO:0045687	6	*Tenm4*, *Pparg*, *Tlr2*, *Il33*, *Serpine2*, *Tnfrsf1b*
	Positive regulation of oligodendrocyte differentiation	GO:0045687	5	*Tenm4*, *Pparg*, *Tlr2*, *Il33*, *Tnfrsf1b*
Cytokine-related	Response to cytokines	GO:0034097	35	*Gpd1*, *Cxcl10*, *Pla2g5*, *Ccl7*, *Was*, *Fosl1*, *Csf1r*, *Il1r2*, *Ciita*, *Sh2b3*, *Ccl2*, *Ifit3*, *Pparg*, *Il1rl*, *Mnda*…
	Cellular response to cytokine stimulus	GO:0071345	30	*Il6r*, *Myc*, *Ccl2*, *Hk2*, *Il1rl1*, *Pparg*, *Sh2b3*, *Gpd1*, *Tfrc*, *Ceacam1*, *Ciita*, *Ptprn*, *Il1r2*, *Tnfrsf1b*, *Il1rl2*…
Cell proliferation-related	Regulation of leukocyte proliferation	GO:0070663	13	*Csf1r*, *Slc39a10*, *Enpp3*, *Mnda*, *Cd28*, *Lrrc32*, *Il33*, *Nfatc2*, *Tfrc*, *Tnfrsf1b*, *Cd55*, *Ceacam1*, *Cd38*
	Regulation of cell population proliferation	GO:0042127	44	*Cxcl10*, *Etv4*, *C5ar2*, *Hmga1*, *Frzb*, *Fosl1*, *Itga2*, *Itgax*, *Areg*, *Csf1r*, *Megf10*, *Scube2*, *Osgin1*, *Slc39a10*, *Enpp3*…

## Data Availability

The data presented in this study are available on request from the corresponding author.
